# Predictors of depression: lifestyle choices during the pandemic

**DOI:** 10.3389/fpsyg.2023.1194270

**Published:** 2023-10-05

**Authors:** Sarka Tesarova, Ondrej Pekacek, Alessandro Porrovecchio

**Affiliations:** ^1^Institute of Sociological Studies, Faculty of Social Sciences, Charles University, Prague, Czechia; ^2^Univ. Littoral Côte d’Opale, Univ. Lille, Univ. Artois - ULR 7369 - URePSSS - Unité de Recherche Pluridisciplinaire Sport Santé Société, Dunkerque, France

**Keywords:** mental health, wellbeing, lifestyle, social media, sport, economic worries, depression, COVID-19

## Abstract

Our study intends to specify the impact of the singular pandemic stressors on the population and also quantify the contribution of different predictors of depression; some of them are stronger than others, and this research shows how the whole effect is divided into single items. This research included a structured online survey using data from 11,340 respondents from six European countries during the first months of the pandemic. The statistical analysis focused on how behavioural patterns appear in different groups of the population and how they mark the psychological wellbeing of these groups with regard to various factors. We targeted social media’s role and analyzed the impact of its consumption on symptoms of depression in different groups divided by age and other characteristics. The analysis creates a mosaic of lifestyle choices and other characteristics that manifest different effects on depression inside selected groups whereas several groups generated by the cluster analysis are less vulnerable to their effect than others. Regarding our findings, the perceived reality through information sources and the manner of their processing seems to be more significant than the tangible reality (poor self-reported health correlated with depression more strongly than intrinsic health limitations).

## Introduction

The outbreak of the novel virus at the beginning of 2020 shook our world and changed our lives in many regards. The quick spread of the disease with a significant mortality rate caused an increasing national and international policy and governance. Lockdown measures were established to stop the spread of the virus and prevent problems with the lack of health care institutions and hospitals’ capacity (Kharroubi and Saleh, 2020). The functioning of services and business was limited or blocked. Many activities such as education and business were put online to limit meeting people and stop spreading the virus. A corollary of these policies was the increasing effect of stressors which had already begun to appear in the public health situation (Brooks et al., 2020). Our global society was facing a lack of expert knowledge about the disease spreading all around the world. This shortage of information created uncertainty regarding which measures were meaningful to prevent the contraction or spread of the virus (Ghebreyesus, 2020). On the backdrop of heightened tension, these circumstances could lead to anxiety symptoms, even depression in some individuals, as someresearchers have already found (Kowal et al., 2020; Mertens et al., 2020). The spread of the new virus COVID-19 also emerged as an information outpouring which is the newly named “infodemic” (Orso et al., 2020). There is no doubt that media and communication technology also play a remarkable role in well-being in these circumstances (Eden et al., 2020). Supposedly, this time represents an extraordinary exposure to the mass media news that shapes people’s opinions and mood and, consequently, their mental well-being (Eden et al., 2020).

## Objective and the study

The role of socio-demographic factors connected with anxiety or depression related to COVID-19 was investigated by a few studies during former years (González-Sanguino et al., 2020; Kowal et al., 2020; Luo et al., 2020; Mertens et al., 2020; Pierce et al., 2020; Vindegaard and Benros, 2020; Wang et al., 2020; Losada-Baltar et al., 2021; Terraneo et al., 2021).

The indicators that can be included as shaping the anxiety or depression in individual personalities are age, parenting (Wang et al., 2020), marital status (Sorokowski et al., 2019)or gender (We use the term “gender” in this paper from the questionnaire. This variable is a part of the socioeconomic status of the informant, so we see it as a social variable). These are common predictors of adding or decreasing the probability of manifesting anxiety symptoms. Other factors could have added intensity to a stressful environment during the pandemic. Self-health rate (SHR) is an index of how personal health is judged by the person (Hossain et al., 2020; Shevlin et al., 2020). Contacts with other people and a particular lifestyle (diet, regular exercise) also affected mental well-being during the pandemic (Asmundson and Taylor, 2020; Mertens et al., 2020).

Looking at the field of well-being, we follow the “Socioemotional selectivity theory,” that explains the reason why age appears to be an important factor that impacts the well-being of people (Carstensen, 2006). People in older age state their inner state of mind as less anxious or depressed than younger people. Certain life stages are connected with more pressure and the longer perspective of living, which creates a different approach to life, which is more focused on the future, creating even more pressure than life itself. This effect shapes a big part of depressive symptoms in a younger part of the population, they anticipate the future more than people who have a shorter perspective on living. A serious illness or circumstance which shortens the life perspective shows the same effect on the symptoms of depression (Carstensen, 2006).

Controversial findings appear in the previous research examining the association between age and depression. As the literature suggests, there seems to be a U-shaped relationship between happiness and age which has been explored and described many times (Blanchflower and Oswald, 2008; Frijters and Beatton, 2012; Laaksonen, 2016; Rauch, 2018). The notion of a U-shape in happiness that well-being is highest for people in their 20s, decreases to its nadir in midlife, and then rises into old age has captured the attention of the media, which often cite it as evidence of midlife crisis (Galambos et al., 2020). The issue of the U-shaped well-being in age is being discussed in detail but also crossed by distinct findings of lately published studies describing decreasing stress levels with age during the pandemic. The defined relationship is not a U-shape as suggested in previous literature but a simpler inverse proportion well-being increases with age (Kowal et al., 2020). Older people report significantly fewer symptoms of depression than young people. Paradoxically, older adults tend to declare worse health conditions while showing lower stress levels and higher well-being than young adults. Generally, stress levels tend to decrease with age and older adults (Bergdahl and Bergdahl, 2002; Archer et al., 2015). Studies generally support the notion that older people are less affected by stressors than younger people (Feizi et al., 2012). Nevertheless, the sources are not consistent, so we decided to rely on the newest findings for claiming our first hypothesis.

*H1*: Higher age is associated with lower levels of depression.

Numerous studies show that women are more susceptible to stress, and they report more anxiety and depressive symptoms than men (Bergdahl and Bergdahl, 2002; Gao et al., 2020). Recent studies confirm the original hypothesis that women experienced more stress during the pandemic than men (Kowal et al., 2020). Thus, we stated our second hypothesis in this sense.

*H2*: Female gender is associated with higher levels of depression.

The variety of factors that can influence anxiety and depression is wide. Living alone is often portrayed as a higher index of depression and anxiety. Dyadic coping is seen as a possible option for the potential shortcomings of being in a relationship, and overall, it seems to provide more benefits than harm (Merz et al., 2014). Families and couples are isolated from the world but in the comfort of their own homes surrounded by friends or relatives. The result of the study shows that married (or cohabiting) individuals experience lower levels of stress than single individuals (Kowal et al., 2020). In general, married individuals are happier, live longer and healthier lives (Lee and Ono, 2012). It is with regards to these sources that we formulated our third hypothesis.

*H3*: People in a relationship experience lower levels of depression.

On the other hand, parenting brings different conditions to the family and elevates stress levels (Abidin, 1990). Many households’ social conditions changed, and people suddenly spent more time in their homes and with their families and children. Parents were expected to deal with homeschooling or school online. The common expectation that parents will be homeschooling their children is associated with being overwhelmed and dissatisfied (Kowal et al., 2020). Parental stress has been associated with numerous negatives and also perceiving the child as difficult (Haskett et al., 2006). Drawing a conclusion from the literature our fourth hypothesis found its shape.

*H4*: Parenthood is associated with significantly different levels of depression.

Understanding the association between education and depression is a part of the wider area which consists of the sociodemographic factors in general and their connection with depression (Schafer et al., 2013). Previous research shows that education, the same as the sociodemographic factors, improves well-being in general and mental and physical health as well (Kowal et al., 2020). These findings shaped the background for our next hypothesis.

*H5*: Higher education is associated with lower levels of depression.

Lack of physical contact with people, in general, has a certain impact on the well-being of people. It is not important if people lost contact with their friends or family members but loneliness during the pandemic appears to be one of the predictors of depressive symptoms even if people do anything to keep themselves preoccupied or distracted (Banerjee and Rai, 2020). The higher level of restrictions due to lockdown measures was associated with more loneliness. Single people who used to have a rich social life were cut off from their friends and social events. A strong correlation between distress caused by a reduction of social contacts and poorer mental health had been previously found (Benke et al., 2020). Considering the results of previous research, we formulated the next hypothesis.

*H6*: Physical contact with friends and family is associated with lower levels of depression.

Alcohol was identified as the most commonly used psychoactive substance (73%) during the SARS-CoV virus pandemic. When regarding alcohol consumption, the study showed that more than 28% of respondents drank to at-risk levels, and almost the same number maintained abstinence (Chodkiewicz et al., 2020). High consumption of alcohol leads to a high amount of cases of depression but the intensity of use seems to be more influential than consumption frequency (Appau and Awaworyi Churchill, 2020).

As the world’s media have publicized preliminary findings suggesting that tobacco consumption can show protective effects against the virus, the higher use of tobacco was also one of the expected results of the pandemic (Alla et al., 2020). On the other hand, the consumption of tobacco is one of the predictors of depressive symptoms (Flensborg-Madsen et al., 2011).

Considering the role of both mentioned influences we created the next hypothesis.

*H7*: Regular consumption of alcohol and tobacco is associated with higher levels of depression.

The expected result on people’s mental well-being was that all kinds of physical activity have a positive effect and lack of that leads to depression (Edwards and Loprinzi, 2016). Several studies suggest that the reduction of total physical activity had a profoundly negative impact on the psychological health and well-being of the population (Maugeri et al., 2020). Physical activity during the pandemic decreased according to a Spanish paper (López-Bueno et al., 2020) but the data from the French dataset analyzed by our research team showed a positive increase in the amount of PA practice (Porrovecchio et al., 2021). The previous findings about physical activities and sports informed the design of our next hypothesis.

*H8*: Regular workouts or physical activity are associated with lower levels of depression.

Physical illness and functional disability are reported as one some of the strongest predictors of problems with a problematic or severe disease course but an even more powerful factor of the psychological well-being of an individual is the subjective approach to personal health, which in the literature is often referred to as “self-rated health.” Expert knowledge about the virus did not seem sufficient, and the expected fear of contracting it or being contagious for other people, especially when the self-rated health is disturbed, is one of the most significant stressors in these circumstances (Kim and Katelyn Kim, 2021). Previous studies identified lower levels of SRH (self-rated health) as an important predictor of symptoms of depression (Kowal et al., 2020; Reigal et al., 2021). According to our expectations and literature we proposed the next hypothesis.

*H9*: Worse self-rated health quality is associated with higher levels of depression.

Economic insecurity, connected with the lockdown rules imposed on entrepreneurs and employees in certain positions or fields (Kowal et al., 2020). Financial loss or economic worries connected with any type of crisis can also be a problem during a quarantine that makes some people unable to work or do their business and the result of a stoppage of their income. In the reviewed studies, the financial loss as a result of quarantine created serious socio-economic distress which suggested an association with depression (Brooks et al., 2020). Our next hypothesis was designed in this sense.

*H10*: Economic distress is associated with higher levels of depressions.

A vital component of our model of a successful strategy in dealing with the pandemic is the influence of media and handling information in general. It is a significant factor that can impact psychological well-being in many regards. Media and technology seem to provide the comfort of contacting loved ones even if they stay at a different place and support anyone with the necessary but also stressful information (Clark et al., 2018). First, it may be an increase in fear and anxiety by publishing too many details and scary news. Secondly, there can also be contradictory messages conveyed by different media with opposite content. Furthermore, it can show possible political, social or economic consequences that can contribute to anxious feelings in society.

Poor information from public health authorities is often seen as a stressor by many people who refer to the deprivation of their liberty as a remarkable source of stress (Brooks et al., 2020). In the case that the authorities fail in transferring adequate information to the public, so-called alternative media takes this role and willingly explains to the public audience all the details which are not provided in the mainstream media or other communication channels from authorities. Consequently, if citizens have little faith in leaders and government institutions to effectively manage a crisis like COVID-19, they might also be less likely to trust government directives and instead turn to alternative sources of information (Crimston et al., 2022). With regards to the previously summarized outcomes, we designed our next hypothesis.

*H11*: Adequate level of public information about Covid-19 transmission and precautionary measures to prevent its spread (handwashing and mask-wearing) is associated with lower levels of depressions.

All sources provided a variety of information about the virus’s medical and biochemical base, statistics about infected and dead people, the situation in other countries, and possible options for protecting against the virus. The information is very often in contradiction; it depends on which source is used. While the mainstream media are churning out the data about lethality and graphs comparing the cases in different countries, the marginal media are speculating about possible distortion in this data using records of experts, doctors, and nurses who conveyed different messages than mainstream, which might cause insecurity that information sources are unreliable, that is reported all over the globe (Rathore and Farooq, 2020; Al-Omoush et al., 2021; Mellado et al., 2021). This feeling of powerlessness can lead to imagining the worst outcome or ‘catastrophizing’, contributing to feelings of anxiety and dread in an already anxiety-provoking period (Wiederhold, 2020). On the other hand, ambiguous or inconsistent media content can fuel thinking about conspiracies and subsequently support stressful factors in the environment (Bruder and Kunert, 2022).

Some sources dismissed the measurements or the medical information about the virus and its manifestation. These are called “conspiracy theories” by some researchers. Daniel Cohnitz claims that these speculations are always rooted in irrational beliefs, which is the basis of this type of thinking (Cohnitz et al., n.d.). Previous studies have also explored the impact of social media on anxiety symptoms (Vannucci et al., 2017). The study revealed a significant association between high use of social media with greater symptoms of anxiety. Subsequent studies have used longitudinal data to examine the impact of watching TV and social media activity on sleeping issues (Tavernier and Willoughby, 2014). Researchers attempted to evaluate the impact of watching TV and social media activity on sleeping issues in a survey in Canada. The media, in general, brings threatening information (e.g., reading news bulletins about new deaths, social media posts), which would increase fear of the virus if it were personally relevant (Stussi et al., 2015). Perceived coping resources may also be predictive of fear of the virus. Coping is a common central mitigating factor in models of health, fear, and pain (Taylor et al., 2020).

Summing up, the problematic role of media exposure in the context of the COVID-19 pandemic should be seen as a multivariate influence on individuals. A wide variety of factors has to be explored in order to bring more knowledge of these effects as frequency/duration of media exposure, type of media, the diversity of media usage and also the content might play an important role in the development of mental distress or might be a result of mental distress (Bendau et al., 2021). On the other hand, many sources are very well informed, and they use conclusions of scientific studies as the foundations for their claims connected with this field.^1^ The public can take a dubious stand against all the information coming from outside because some of these sources are taken away without a credible explanation, or the explanation has a low quality of argument (Orso et al., 2020). This battle between the mainstream and alternative media can also fuel stress levels among the public audience. Social media is commonly referred to as a stress-increasing factor. However, it is also used as a substitutional virtual contact with friends and family instead of living contacts (Banerjee and Rai, 2020). We reflected upon the complex media situation in our last hypothesis.

*H12*: Use of social media is associated with higher levels of depression.

## Methods

### Participants and procedure

This research project is based on the umbrella project “Pandemic Emergency in Social Perspective. Evidence from a large Web-survey research,” designed and organized by principal investigators Linda Lombi (Università Cattolica del Sacro Cuore, Milan) and Marco Terraneo (Università Bicocca-Milano). This research gathered data in seven European countries (Italy, Sweden, France, CR, Spain, UK, Poland) in March 2020. The data collection was realized during the pandemic lock-down in the period 27.3.2020–10.6.2020. The international team used the convenient data collection approach and reached the informants on social networks. All teams created separated links to our survey website with the different language versions. After the final cleaning we gained 9,541 cases in all countries The results of this research were already published in the following articles (Porrovecchio et al., 2021; Terraneo et al., 2021).

### Measures

The principal goal of the international cross-sectional study is to explore how the sanitary emergency due to the COVID-19 pandemic is affecting peoples’ habits, lifestyles, and psycho-physical well-being to uncover any existing problems and try to propose solutions necessary to achieve greater well-being among the community.

This study focuses primarily on the predictors of depression within the European context of the Covid-19 pandemic, specifically during the lockdown and social distancing period of March–April 2020. Our team decided to explore the impact of behavioral/lifestyle factors that are modifiable, such as exercise, alcohol or social contacts but also the usage of media, mainly social media as a source of information about the situation in the world.

Our hypotheses are based on literature review, and we also used the Czech sample of the whole dataset as a test part for testing our hypotheses and we have confirmed them with the analysis on the international dataset. In order to have a clear record about our hypotheses and decisions at the beginning of our research, we created the pre-registration at OSF platform. The record is visible here: https://osf.io/yx7sr.

### Statistical analyzes

All statistical analyzes were performed using R (version 4.0.2). A value of p of <0.05 was considered statistically significant. We performed standard descriptive statistics with a correlation matrix between individual predictors based on the Spearman coefficient. Before we started analyzing regression models, we tested several stepwise and literature-based models’ performance.

A detailed view of our sample and figures, charts and source code of our computation is available here: http://covid19.saturnin.info/wp-content/uploads/2022/03/Covid_International_Sample_Report.html.

Regarding our interest in specifying how single predictors influence mental well-being in different combinations and how strong they are, we performed regression analysis with various result models.

Results of our regression analysis are available here: http://covid19.saturnin.info/wp-content/uploads/2022/03/2.multiple_regression_adjusted_contrasts.html.

In order to define specific groups with distinct characteristics, behavior and vulnerability, we used cluster analysis and tested the relevance of extracted groups.

Visualization and computation results of our cluster analysis are available here: http://covid19.saturnin.info/wp-content/uploads/2022/03/3.Clustering_visualizations.html.

Collection of the data was designed as an internet questionnaire; therefore we have to deal with the representativity of the sample. The internet-based surveys are often labeled as non-representative (Babbie, 2010) but the most important point of our study was a quick response to the situation. The online survey enabled us to obtain answers quickly in a short time period when the situation was exacerbated and unique in its circumstances. Still, the form of how we combined two types of getting answers also improves the representativity of the survey. We also count on using the datasets from other countries for checking if the patterns will appear in the different countries, and then we can consider the findings as generally valid (Babbie, 2010).

We can see some resemblance between the contemporary situation and September 11th, 2001. The circumstances have been changing quickly and unpredictably; people felt surprised and drawn into the topic. That was why we needed to take action quite rapidly, and there was not much time to prepare the methodology of data collection. We collected data when people were interested because it was “a topic of today.” We see this project as an opportunity for using the same methodological approach as the researchers in 2001 used (Jerabek and Veisova, 2001).

## Results

The correlation matrix based on the Spearman coefficient turned out as the suitable first step in the analytic process. Even though this coefficient is not suitable for most of the variables, because they are not cardinal, it can be used as the first indicator for next analytic steps, because the most visible association can be revealed in this manner and the usage is meaningful for getting a simple sign if the variable can be included into the next analysis. The only cardinal variable in the dataset is age and we already see in this step a significant negative correlation between variable PHQ8 and age that is in accord with the findings in the previous research in this field. There is also visible association between type of relationship, self-reported health, consumption of social media and the variable PHQ8, that is the first sign about possible role of these variables in our model and it is an indicator for placing them into the next analysis. See: 1 Correlation plot (Spearman, multiply imputed dataset) – Overview of correlations between individual predictors and outcome.

The next step in the analysis was creating a regression model, which we found a suitable approach for detailed exploration of associations between known variables and symptoms of depression. The first model as a background for our hypotheses was generated with data in the Czech data set, and we created the expected results based on this analysis. The final output of the regression model was counted from the international dataset (the Czech part was not included). The complex regression model required creating the specific cumulative variable PHQ8, which is the dependent variable intended to determine the presence and severity of the major depressive disorder. This construction is standardized and based on the established methodology (Kroenke et al., 2009), See: 1.2.1 Sample descriptive statistics.

### Regression models

We compared results of two types of regression models, the first was inductively built according to the literature findings, and we have been gradually adding the following predictors suggested by the literature, and then we can see how the models have changed after this adjustment. Our analytic steps started with the simple model, which used only demographic characteristics as predictors for depression, and we have been adding the following variables gradually.

The second approach to creating models is “stepwise” built models which are calculated by optimizing the results of specific associations between the predictors in our dataset. The process of building the stepwise model was computed by the specific statistical algorithm created for this purpose. The algorithm generated two models, the first included the basic sociodemographic (Table 1). Drawing on two methodological approaches, we created two models. The first one was built according to Bayesian Information Criteria (BIC), which puts a larger penalty on the complexity of the model, and the second (Model 2) we created by using the Akaikes Information Criteria (AIC) method, which is usually assumed to be more suitable when the goal is prediction. As we can see, the most suitable models are Stepwise AIC model (est 0.229) (Table 2) and Theory-derived Model 4 (est 0.235) (Table 3). The difference between both coefficients is very small and we should take both models into account as equivalent sources of interpretation. See: 2.1 Model fit measures.

**Table 1:** Stepwise BIC Model: ANOVA test.

	SSQ	df1	df2	*F* value	Pr (>F)	eta2	partial.eta2
q02_age	9,503	1	22,089	404.8	0	0.040	0.049
q47_self_reporting_health	25,875	4	673	265.0	0	0.109	0.123
q36_econ_worry	10,486	2	8,171	222.3	0	0.054	0.054
q01_gender	4,246	2	4,005	89.3	0	0.022	0.022
q18_02_soc_media	1,072	1	91,553	46.0	0	0.006	0.006
q49_health_limitations	869	2	818	17.6	0	0.005	0.005
q04_children	377	1	103,489	16.1	0	0.002	0.002
Residual	184,759	NA	NA	NA	NA	NA	NA

**Table 2:** Stepwise AIC Model: ANOVA test.

	SSQ	df1	df2	*F* value	Pr (>*F*)	eta2	partial.eta2
q02_age	9503.34	1	18,588	408.006	0.000	0.040	0.049
q47_self_reporting_health	25874.63	4	604	266.660	0.000	0.109	0.124
q36_econ_worry	10486.26	2	6,967	223.990	0.000	0.044	0.054
q01_gender	4246.21	2	3,533	89.943	0.000	0.018	0.023
q18_02_soc_media	1071.97	1	82,074	46.374	0.000	0.005	0.006
q20_public_info	1143.97	1	217	42.881	0.000	0.005	0.005
q49_health_limitations	868.79	2	785	17.745	0.000	0.004	0.002
q04_children	376.57	1	99,272	16.295	0.000	0.002	0.003
q03_relationship_type	589.98	4	2,198	6.204	0.000	0.002	0.001
q38_alcohol	128.50	1	95,452	5.553	0.018	0.001	0.000
q40_smoking	24.16	1	2,709	0.954	0.329	0.000	0.000
q34_02_face_mask	12.64	1	5,292	0.494	0.483	0.000	0.000
q48_chronic_illness	1.87	1	2,207	0.014	0.907	0.000	0.000
Residual	182858.38	NA	NA	NA	NA	NA	NA

**Table 3:** Theory-derived Model 4: ANOVA test.

	SSQ	df1	df2	*F* value	Pr (>*F*)	eta2	partial.eta2
q02_age	9503.34	1	19079.2	410.962	0.000	0.040	0.050
q47_self_reporting_health	24247.04	4	1293.9	256.189	0.000	0.102	0.118
q36_econ_worry	8744.33	2	4604.9	187.230	0.000	0.037	0.046
q20_public_info	3052.33	1	66.0	100.597	0.000	0.013	0.017
q01_gender	4246.21	2	3783.7	90.664	0.000	0.018	0.023
q18_02_soc_media	1320.21	1	211780.2	57.676	0.000	0.006	0.007
q35_01_contact_close_family	1289.09	2	2545.1	27.325	0.000	0.005	0.007
q42_sport	381.22	1	1013.5	15.585	0.000	0.002	0.002
q03_relationship_type	1357.12	4	6950.9	14.629	0.000	0.006	0.007
q49_health_limitations	679.95	2	323.0	13.435	0.000	0.003	0.004
q40_smoking	153.29	1	2164.7	6.373	0.012	0.001	0.001
q11_education	405.70	2	28.1	5.719	0.008	0.002	0.002
q34_07_hand_washing	127.14	1	4039.7	5.357	0.021	0.001	0.001
q48_chronic_illness	76.45	1	95853.1	3.323	0.068	0.000	0.000
q04_children	6847.00	1	54036.4	2.966	0.085	0.000	0.000
q35_03_contact_friends	118.51	2	1466.8	2.426	0.089	0.000	0.001
q34_02_face_mask	20.67	1	18810.9	0.872	0.350	0.000	0.000
q38_alcohol	0.45	1	95005.8	0.010	0.921	0.000	0.000
Residual	181395.71	NA	NA	NA	NA	NA	NA

The three most influential predictors for depressive symptoms are the same in both tables. The essential modulator of depression is AGE with the *f*-values in both models F (408|410). The predictor SRH (self-reported health) also shows a substantial impact on symptoms of depression as the second in a row F (266|256), and economic worry is the third influential factor for depression F (223|187). The following items seem to be different in both models. The “stepwise” model orders the strength of single factors in this order: gender, social media, public info, health-limitation, children, relationship type, whereas the theory derived model shows a different order: public info, gender, social media, contact close family, sport, relationship type, health-limitations, smoking, education and also hand-washing.

As we can see in our regression models, more than four effects rise into the foreground with a salient effect on depression. First of all is age, which is an outstanding factor that affects depressive symptoms; the indirect proportion between these two variables is observable, younger people are more susceptible to depression than older people, and a poor subjective perception of personal health is also a powerful predictor for depressive symptoms. This predictor shows co-functioning with the reports of health limitation and possible chronic illness because these factors are strongly correlated, but the subjective perspective appears to be the most important of all connected variables. Economic worries are detected as a significant predictor of depression in both models, and it seems to be a more substantial influence than gender. We also found other detectable influences, as consumption of social media is a significant predictor for raising symptoms of depression, and also people who felt a lack of information from the public sources were more depressed than others. Decreased frequency of contact with close family also shows a significant negative impact on the symptoms of depression, people who met their close relatives less often than before the emergency state seem to be more vulnerable to depression and the counting of their symptoms raised.

The analysis proved some of our hypotheses, and some of them remained unconfirmed. We proved that the strongest influence on depression is age, the younger people are more susceptible to depression than older people. We confirmed the hypotheses that worse self-rated health, economic distress, female gender, higher consumption of social media, decreased physical contact with friends and family, less physical activity and single status are associated with higher rates of depressive symptoms. On the other hand, our analysis did not prove the other hypotheses that parenting, higher education, smoking and alcohol consumption increase symptoms of depression.

The report with all the regression model analysis is available here: 2 Regression models (multiply imputed dataset).

### Cluster analysis

**Figure 1:**
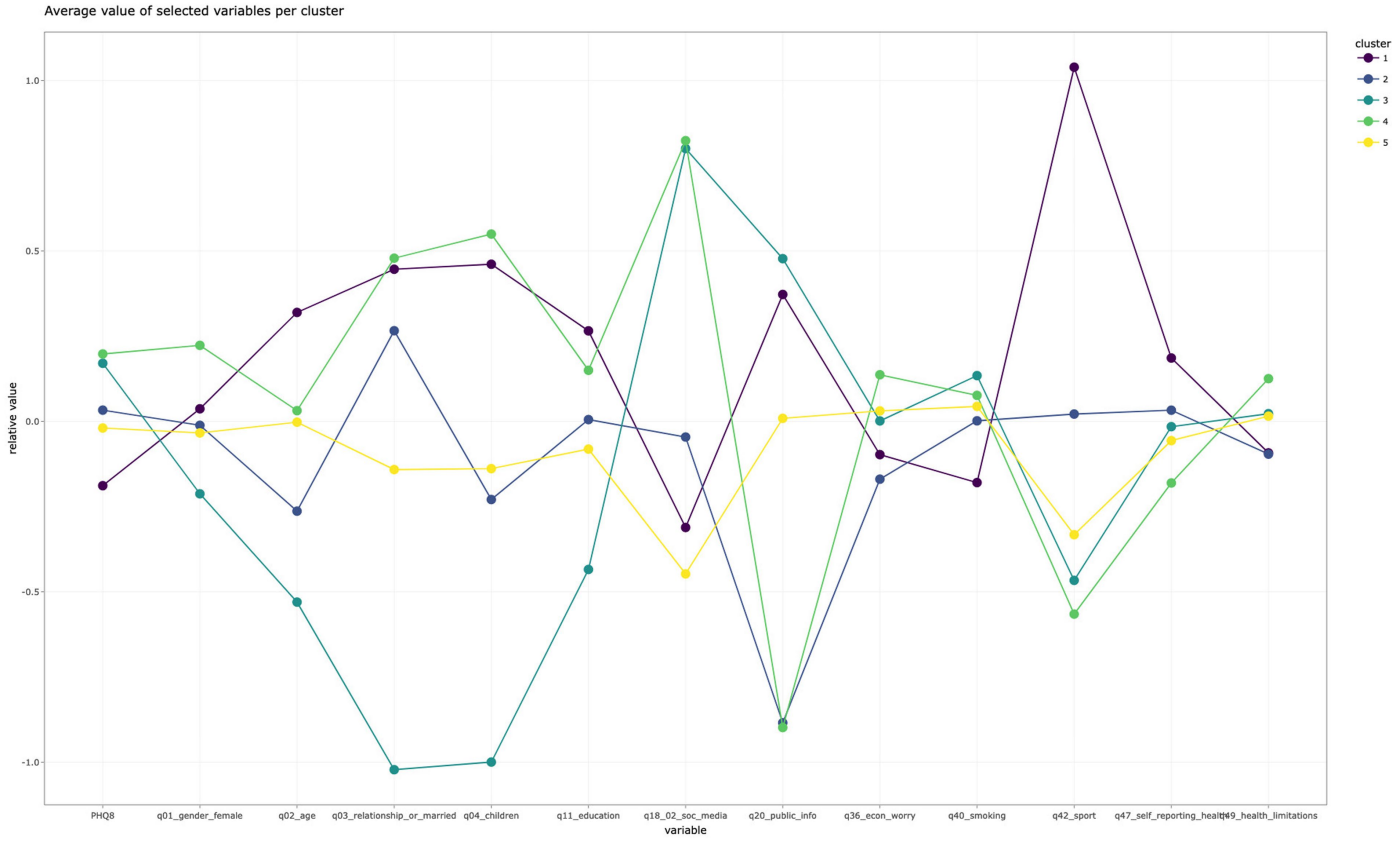
Visualization – cluster analysis.

We are aware of the limitations of the regression analysis, which approaches the dataset as one homogeneous group and tries to find the most important influences in this quite heterogeneous environment; thus, we decided to use K-medoid analysis to find groups with similar characteristics as an extensive descriptive option. We conducted clusters according to the algorithm, the so-called “elbow-method,” which shows a graph with a gradually rising depth of information with each division for more groups. We have more group options computed; then we had an opportunity to see that the low number of groups in the model (two or three groups) does not distinguish specific groups too closely and conversely, division in too many groups leads to obfuscating results. So, finally, we decided to choose the “five-group K-medoid analysis,” which seems to be the optimal division that shows single clusters and manifests their specific characteristics (Figure 1).

The five-group K-medoid cluster divides the dataset into five groups, The first eye-catching divider is the variable sport in the “purple” group, which shows a remarkable difference in group 1 (purple), which gathers people who are the least depressed than other groups. The purple group has the highest age, people in this group are often married or in a relationship and have children, they perceive themselves as well informed, they usually do not smoke, and they manifest the highest rate of physical activity (sport) and also the best health of all other groups. These people most likely are not involved in social media, and their education is the highest of all, and this group includes both genders almost equally.

The “green” group can be tracked by health limitations and poor health in general. This group is older than average, education also higher than average and most likely women with a high frequency of depression. This group’s social media consumption is higher than average, but the personal feeling of “being informed” is the lowest one. These people are most likely married with children, smoke more often than average, and have the highest rate of economic worries. Almost the same level of depression shows the “light blue” group, which consists mainly of younger people who are primarily single without children, very often lacking sports activity but their consumption of social media is high, but they also feel themselves well-informed. The “dark-blue” group has quite good health without limitations. They are usually in a relationship without children and feel well informed. The “yellow” group are people in their middle age who usually do not use social media and do not do any sports activity, and their depression is relatively moderate; their rate is below average.

The report with all the cluster analysis Visualization is available here: K-Medoids Clustering: Cluster Visuals.

## Discussion

Our study intended to describe the most influential factors for depression during the pandemic, as in many other studies. However, they usually focus on one single influence in detail or investigate more influences by measuring their strength. On the contrary, we claimed a goal to analyze the volume of influences in their combination, extract groups with distinct characteristics, and describe the individual combination of factors for each. We aimed to answer our hypotheses by multiple regression models. We have found significant connections, but they do not appear to have the same relevance for all cases in our dataset.

So, we decided to use cluster analysis and find groups influenced by different factors, as suggested in other studies (Kowal et al., 2020). The three most important factors influencing depression during the pandemic were age, health issues, and economic worries. Our analysis shows an almost clear negative correlation between depressive symptoms and age (the higher the participant’s age, the lower the symptoms of depression appear). On the other hand, a recent study (Zaninotto et al., 2022) revealed that the prevalence of clinically significant depressive symptoms increased in the group of older adults, which is in contradiction with our findings. This difference may be caused by the different times when the data was collected, and their results include the primary impact of the substantial change. In contrast, our data was collected later when people started to get used to the new situation. At the same time, this difference shows how important it is to examine individual factors in their mutual interaction and thus discover more profound connections between predictors and their actual outcomes.

We can also say that lifestyle and social media consumption are salient factors for depression, but the relationship is not simple; some groups are more influenced than others. Our results support the previous findings that social media can be used in more different ways without positively or negatively impacting their consumers (Radovic et al., 2017). The positive aspects of online activities can be promoted, but also detrimental effects or addictive media use may appear more frequently in the pandemic or social isolation (Marciano et al., 2022).

The cluster analysis reveals the more sophisticated relationships between these factors and depression.

As we can see from our cluster chart, the purple group is characterized by higher age, good health and regular exercise and the lowest rate of depression. These people also do not perceive social media as the first source of their information, and they feel well-informed. On the contrary, we can also see another trend (green group) with higher age but without physical activities, with health issues and increased consumption of social media as the group with the highest rate of depression. Comparing these two groups, we can see that the age without other cooperative factors does not change anything and is not supportive as it is proposed in previous research (Feizi et al., 2012; Kowal et al., 2020).

We can also see a younger group (light blue) with a high depression rate as frequent social media consumers and without sports activities. The two most depressed groups of the chart have one common denominator: social media and lack of physical activity. We can identify these two groups as having similar lifestyles varying in different age groups; the younger is usually single and without children, and the older is the opposite. However, their rate of depression is almost the same, and we can say that their lifestyle is similar. Despite their different socioeconomic characteristics, the results are similar, supporting previous findings that sedentary behavior is more likely associated with depression (Porrovecchio et al., 2021). It is tempting to see physical activity as the straightforward prevention against depression. Still, the other chart with six clusters (more detailed), reveals a group which contains mainly younger people who exercise regularly. However, their rate of depression is still higher than average.

As we proposed before, the second most influential factor in building depression, according to the results in regression models, is self-rated health and health limitations; these are strongly correlated, which is in agreement with the findings of the previous study (Terraneo et al., 2021). This presumption is visible in the five clusters chart. Still, the six clusters chart reveals that the youngest group (cluster 4) is the most depressed group despite its health being only slightly below average. Thus we can distinguish the influence of age as more forcible than the role of self-rated health; even public media served the picture of old and unhealthy people as the most endangered people by the infection of the virus (Gallagher, 2020). It is supposed to lead to a higher rate of depression among this vulnerable population (Wheaton et al., 2021).

We can also speculate about the role of public information while seeing a moderate depression in the six clusters chart, which contains people with higher age, higher education, and some health issues who do not feel well informed (yellow cluster). The feeling of lack of information, despite the media not reporting anything other than the pandemic, can be caused by the media’s emotional content, which this group does not perceive as reliable enough (Wheaton et al., 2021).

Social media is undoubtedly a divider among groups in the six clusters chart, which shows that the two most depressed groups use social media as their primary source of information. We can estimate that the association between depression and the use of social media as a source of information can be tainted by emotionally contagious content with a strong impact on depression (Wheaton et al., 2021). We can also speculate about the role of social media’s personal content in groups revealed in the latest research. The first group is stressed because of the fear of the disease and the second is stressed because they see the fear of the virus as deliberately exaggerated. They are more afraid of societal changes associated with inadequate measures (Taylor et al., 2020).

Economic worries are one of the most influential factors; they do not seem to be a division of single groups; we can say that financial worries are a salient independent effect across the whole population. We have not found a single group with a high peak or drop of economic worries.

### Limitations

This study is associated with limitations connected with online surveys. Our team considered the situation exceptional and it was necessary to obtain data quickly and according to relevant methodological literature (Babbie, 2010) is our sample valid for the used type of analysis. Detailed explanation is described in the Methodological chapter.

The authors are also aware of the limitation of excluding gray and non-peer-reviewed literature from this paper. The amount of peer-reviewed literature towards this topic is sufficient.

## Conclusion

According to the results of our analysis, we see areas where there can be intervention to improve the situation. People affected by health issues are already a vulnerable part of the population because of their illness. Still, their social media consumption probably does not help them improve their well-being. The same conclusion can be made about the young population who seem to be the most vulnerable to depression and are the highest consumers of social media. The other research has already designed the solution to the problem with social media consumption, which debunked more manners of social media use. People can be taught how to use social media to help them improve their mental health and not to destroy it (Haris et al., 2014). The second possible intervention can be proposed to the specific group with the highest education in the whole sample as a higher level of public information (Taylor et al., 2020). Regarding the high level of education in this group, we suppose that the information provided during the pandemic by the authoritative sources was insufficient or not sophisticated enough to satisfy these people. Both cases of a possible intervention should be investigated in more detail in more focused qualitative research, revealing the best way to deal with these problems.

## Data availability statement

The raw data supporting the conclusions of this article will be made available by the authors, without undue reservation.

## Ethics statement

The study was approved by the Ethics Committee of the Policlinico Universitario A. Gemelli IRCCS of Università Cattolica del Sacro Cuore (prot. 0025523/20).

## Author contributions

ST contributed to the main research question, carrying out the literature search, collecting the included studies information, writing, and describing the results. OP worked on data curation, conceptualization, formal analysis, and visualization. AP contributed with supervision and validation. All authors contributed to the article and approved the submitted version.

## Footnotes

^1^ swprs.org
