# Supplementary figures and images for: Predictors of depression: lifestyle choices during the pandemic
Source: Front Psychol. 2023 Oct 5;14:1194270. doi: 10.3389/fpsyg.2023.1194270 (PMC10585652; doi:10.3389/fpsyg.2023.1194270)

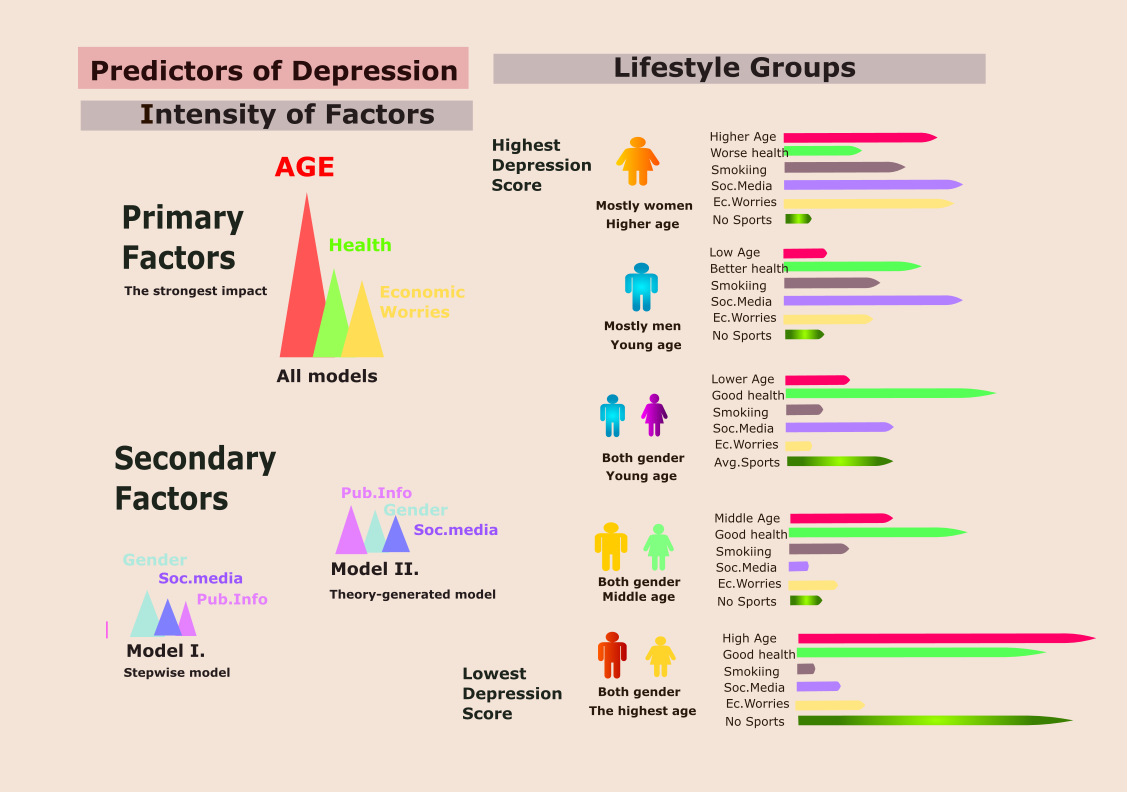

Supplement: Supplementary file 1 [file Image_1.PNG]
